# Anticipatory syringe pumps: benefits and risks

**DOI:** 10.1136/bmjspcare-2020-002735

**Published:** 2021-01-19

**Authors:** Ben Bowers, Kristian Pollock, Andrew Dickman, Richella Ryan, Stephen Barclay

**Affiliations:** 1 Department of Public Health and Primary Care, University of Cambridge, Cambridge, Cambridgeshire, UK; 2 Nottingham Centre for the Advancement of Research into Supportive, Palliative and End of Life Care, School of Health Sciences, University of Nottingham, Nottingham, Nottinghamshire, UK; 3 Academic Palliative and End of Life Care Centre, Liverpool University Hospitals NHS Foundation Trust, Liverpool, UK; 4 Arthur Rank Hospice Charity, Cambridge, Cambridgeshire, UK

**Keywords:** clinical assessment, drug administration, home care, nursing home care, symptoms and symptom management, terminal care

We welcome the Association of Supportive and Palliative Care Pharmacy’s (ASPCP) recent position statement that the perceived benefit of the anticipatory prescribing of a syringe pump (driver) does not outweigh potential risks.^1^ This is an area of practice which has needed clear national guidance for some time.^2^

The committee cited the following specific risks:

1. A lack of individualisation.
2. No anticipation of dose/drug changes between prescribing and initiation.
3. Administration errors.^1^

Given these risks, it is of concern that anticipatory syringe pump prescribing appears relatively common in some areas of the UK and is perhaps increasing during the COVID-19 pandemic.^3^

Population-level information is lacking on how many patients are prescribed or commenced on syringe pumps at home, although they are common in generalist community palliative care.^4 5^ Immediately pre-COVID, we completed a retrospective observational study of anticipatory medication practice in primary care (paper submitted for publication). We reviewed 329 patient records from 11 general practitioner (GP) practices (30 most recent predictable deaths per practice) in two English counties. Anticipatory syringe pumps were prescribed for 49/167 (29.3%) patients issued with anticipatory medications, with considerable variation in frequency between GP practices, ranging from 10/14 patients (71.4%) to 1/16 patients (6.3%) across practices. Prescription timing varied from 536 to 0 days before death (median 5.5). There were notably diverse prescribing cultures: median time from prescription to death ranged from 27 to 2 days across individual GP practices. Anticipatory syringe pumps were only commenced for 22/49 (44.9%) of those for whom they were prescribed and authorised. In four cases, anticipatory syringe pump prescriptions were in the home for more than 3 months before the patient’s death, and in only one of these cases was the syringe pump actually commenced. Our data suggest potential safety issues in the timing and appropriateness of some prescribing practices.

There is a high chance that prescription needs will change over a period of 1–3 months. Drugs prescribed far in advance are unlikely to meet current needs in an individualised way. Advance prescriptions of several drugs and wide dose ranges via a syringe pump may encourage initiation without a sufficiently detailed skilled assessment.^2 6 7^ Our recent review of a random sample of 28 local community anticipatory prescribing policies and drug authorisation charts in England found explicit guidance regarding the appropriateness of anticipatory syringe pumps was in place in only 12 areas (paper in preparation). This suggests unregulated practice exists in some areas where anticipatory syringe pumps are used, without robust governance and documentation systems to support practice. There is a real risk of administration errors in the absence of reminders and checks to ensure oral medicines are stopped, and existing transdermal medicines are considered, when anticipatory syringe pumps are commenced.^1^

There may well be individual cases when an anticipatory syringe pump is appropriate when a patient has unambiguous and pre-existing symptom control needs. Howard *et al* 8 helpfully proposed the following criteria:

1. Patient circumstances are foreseeable and unambiguous even for those without specialist experience.
2. Medication choices and doses are unlikely to change.
3. Initiation of the syringe pump is reliably followed by a timely review by a skilled clinician.

This would apply, for example, when someone is already on high-dose oral opioids or an oral anticonvulsant and a plan is needed for an imminent situation when they are no longer able to take these medications. The individualised prescribing of an anticipatory syringe pump after specialist palliative care advice would offer timely access to continuous medication at this stage, but in our view, these are relatively unusual occurrences.

The above prescribing and administration conditions are even tougher to achieve during the COVID-19 pandemic, with reported increases in remote prescribing and reductions in GP and specialist home visits.^3^ Anticipatory syringe pumps must not become routine medical practice^9^ regardless of how tempting it is to prescribe them alongside *pro re nata* (PRN—as needed) injections as an additional security blanket.

Prescriptions should be based on an assessment of individualised care needs, and with the agreement of the patient, wherever possible, and family caregivers.^6 10^ This involves a suitably skilled prescriber assessing likely needs and responding sensitively to patient and family questions regarding the purpose of the drugs and a syringe pump.^4 10^ Current evidence suggests these conversations may be cursory and patients may have limited awareness about anticipatory medications, including anticipatory syringe pumps.^7 11^ We are currently researching patient and family caregiver views and preferences regarding anticipatory prescribing including pumps.

There is a danger specialist palliative care practice for complex needs are being followed in general practice for persons with less complex symptom profiles. Concurring with the ASPCP, we recommend syringe pumps should only be prescribed after a face-to-face clinical review by a skilled prescriber to consider causes of deterioration and associated symptoms, evaluate reversibility, establish a dying diagnosis and appraise the effectiveness of previously administered oral and PRN drug injections. In the relatively unusual circumstances in which an anticipatory syringe pump is appropriate, it is important to ensure that prescribers regularly reassess the patient’s needs and review the prescription accordingly.
